# The role played by cell-substrate interactions in the pathogenesis of osteoclast-mediated peri-implant osteolysis

**DOI:** 10.1186/ar1938

**Published:** 2006-04-13

**Authors:** Zhenxin Shen, Tania N Crotti, Kevin P McHugh, Kenichiro Matsuzaki, Ellen M Gravallese, Benjamin E Bierbaum, Steven R Goldring

**Affiliations:** 1 New England Baptist Bone and Joint Institute, Beth Israel Deaconess Medical Center, Harvard Medical School, Boston, Massachusetts, USA; 2 Department of Orthopaedic Surgery, Beth Israel Deaconess Medical Center, Harvard Medical School, Boston, Massachusetts, USA; 3 Department of Orthopedics, New England Baptist Hospital, Boston, Massachusetts, USA

## Abstract

Prosthetic wear debris-induced peri-implant osteolysis is a major cause of aseptic loosening after total joint replacement. In this condition, wear particles released from the implant components induce a granulomatous inflammatory reaction at the interface between implant and adjacent bone, leading to progressive bone resorption and loss of fixation. The present study was undertaken to characterize definitively the phenotype of osteoclast-like cells associated with regions of peri-implant focal bone resorption and to compare the phenotypic features of these cells with those of mononucleated and multinucleated cells associated with polyethylene wear particles. Peri-implant tissues were obtained from patients undergoing hip revision surgery for aseptic loosening after total joint replacement. Cells were examined for the expression of several markers associated with the osteoclast phenotype using immunohistochemistry, histochemistry, and/or *in situ *hybridization. CD68 protein, a marker expressed by multiple macrophage lineage cell types, was detected in mononucleated and multinucleated cells associated with polyethylene particles and the bone surface. Cathepsin K and tartrate-resistant acid phosphatase were expressed highly in both mononucleated and multinucleated cells associated with the bone surface. Levels of expression were much lower in cells associated with polyethylene particles. High levels of β_3 _integrin protein were detected in cells in contact with bone. Multinucleated cells associated with polyethylene particles exhibited faint positive staining. Calcitonin receptor mRNA expression was detected solely in multinucleated cells present in resorption lacunae on the bone surface and was absent in cells associated with polyethylene particles. Our findings provide further evidence that cells expressing the full repertoire of osteoclast phenotypic markers are involved in the pathogenesis of peri-implant osteolysis after total joint replacement. They also demonstrate that foreign body giant cells, although believed to be phenotypically and functionally distinct from osteoclasts, express many osteoclast-associated genes and gene products. However, the levels and patterns of expression of these genes in the two cell types differ. We speculate that, in addition to the role of cytokines and growth factors, the substrate with which these cells interact plays a critical role in their differential phenotypic and functional properties.

## Introduction

Inflammatory processes that target the skeleton are frequently accompanied by a localized disturbance in bone remodeling. The present study investigates a prototypical inflammatory disorder, namely peri-implant osteolysis after total joint replacement (TJR), in which localized bone resorption ultimately leads to loss of prosthetic fixation and implant loosening. In this condition, wear particles generated from orthopaedic implant components or from bone cement used for fixation gain access to the peri-implant bone interface, where they induce a granulomatous inflammatory reaction characterized by the presence of fibroblast-like cells, macrophages, and multinucleated foreign body giant cells. In localized areas where the inflammatory tissue is in contact with the bone surface there are focal regions containing mononucleated and multinucleated 'osteoclast-like' cells residing within resorption lacunae. These osteoclast-like cells have been implicated in the pathogenesis of the bone resorption associated with peri-implant osteolysis. Takagi and coworkers [1] demonstrated high-turnover peri-prosthetic bone remodeling and immature bone formation around loosened total hip replacement implants, indicating that the key role for the peri-implant osteoclast is in peri-implant bone resorption.

The macrophages, multinucleated foreign body giant cells, and osteoclasts that are present within the peri-implant tissues are derived from a common hematopoietic lineage, and a variety of phenotypic markers have been utilized to distinguish these cells from each other. Included among these are a variety of genes and gene products that impart to the osteoclast the unique capacity to recognize and bind to the bone surface in order to resorb a mineralized bone matrix. The attachment and activation of the osteoclast has been shown to involve several different integrins, including the vitronectin receptor α_v_β_3 _[2,3]. Expression of this integrin has served as a useful marker to identify osteoclasts and to distinguish them from their colony forming unit-macrophage (CFU-M) precursors that do not express the β_3 _gene [4]. Additional gene products that are essential for creating an acidic environment for mineral dissolution and resorption of the organic matrix of bone are induced during osteoclast differentiation. Cathepsin K and tartrate-resistant acid phosphatase (TRAP) are among the enzymes that are expressed in these cells and contribute to the resorption of the extracellular matrix component of bone [5-7].

Although the expressions of these genes have served as useful markers to identify osteoclasts, several studies have demonstrated that their expression is not restricted to osteoclasts. For example, under certain conditions, TRAP activity and cathepsin K have been detected in cells that are not involved directly in bone resorption [8-10]. In our own studies involving analysis of synovial tissues from patients with rheumatoid arthritis [11] we observed that, in addition to cathepsin K and TRAP expression, osteoclast-like cells in resorption lacunae at the bone-pannus interface express the calcitonin receptor (CTR). In *in vitro *mouse and human osteoclast differentiation models, expression of the CTR occurs during the terminal stage of osteoclast differentiation, and activation coincides with the competence of the cell to resorb bone. The expression of this gene and gene product can thus be used to help discriminate mature osteoclasts from macrophages or macrophage polykaryons, and to identify osteoclasts that are actively involved in bone resorption.

In the present study we utilized immunohistochemical, histochemical and *in situ *hybridization techniques to analyze the phenotype of cells in human peri-implant tissues from patients with aseptic implant loosening after TJR. Special attention was focused on the differential phenotype of cells associated with polyethylene wear particles or the bone surface. Our results provide further evidence that cells expressing the full repertoire of osteoclast phenotypic markers are involved in the pathogenesis of peri-implant osteolysis after TJR. They also demonstrate that foreign body giant cells, although believed to be phenotypically and functionally distinct from osteoclasts, express many osteoclast-associated genes and gene products. However, the levels and pattern of expression of these genes in the two cell types differs. Osteoclasts and foreign body giant cells are derived from a common hematopoietic precursor, and we speculate that, in addition to the role of cytokines and growth factors, the substrate with which these cells interacts plays a critical role in their differential phenotypic and functional properties.

## Materials and methods

### Human tissue collection and preparation

Human peri-implant tissues associated with foreign body reactions to orthopedic implant wear debris were obtained from 12 patients. These patients had a clinical history of osteoarthritis and were undergoing revision surgery for aseptic loosening of prosthetic components after total hip replacement. The patients' ages ranged between 45 and 87 years; nine were female and three were male. Patients with a prior history of inflammatory arthritis were excluded from the analyses. The study protocol was approved by the New England Baptist Hospital and the Beth Israel Deaconess Medical Center Institutional Review Boards, and informed consent was obtained from all patients before surgery.

Specimens of soft tissue and bone were collected from regions of bone resorption during joint revision surgery. The specimens were fixed in freshly prepared 4% paraformadehyde, followed by demineralization with 14% EDTA in phosphate-buffered saline (PBS). The specimens were processed and embedded in paraffin and 5 μm sections were prepared for histological, histochemical, and immunohistochemical analyses.

### Reagents for immunohistochemical detection and probes for *in situ* hybridisation

Antibodies included a rabbit polyclonal antibody to human CD68 (sc-9139; Santa Cruz Biotechnology Inc., Santa Cruz, CA, USA), which identifies macrophages and osteoclasts, and a goat polyclonal antibody to human β_3 _integrin (sc-6627; Santa Cruz Biotechnology Inc., Santa Cruz, CA, USA). A rabbit polyclonal antibody to human cathepsin K was kindly provided by Dr D Bromme. The ABC avidin-biotin-peroxidase complex kits were purchased from Vector Laboratories (Burlingame, CA USA). RNA antisense probes for cathepsin K, TRAP, and CTR were prepared as previously reported [11,12] and sense probes were used as negative controls.

### Histochemistry

Histochemical staining for TRAP activity was done as previously reported [11]. The sections were incubated with the reagents at 37°C for 10–20 minutes followed by counterstaining with hematoxylin.

### Immunohistochemistry

For immunohistochemistry, sections were dewaxed and subjected to antigen retrieval in 10 mmol/l EDTA (pH 7.5) and microwaved at 93°C for 7 minutes. Immunohistochemical staining was performed as previously reported [13]. Briefly, after rinsing with PBS the sections were pretreated with 3.0% hydrogen peroxide at room temperature for 20 minutes to inhibit endogenous peroxidase. Sections were then treated with blocking solution containing 1.5% (vol/vol) normal goat or rabbit serum (based on the animal secondary antibodies) and 10% fetal calf serum for 60 minutes at room temperature. Excess serum was gently blotted off and the sections were incubated with primary antibodies diluted in PBS containing 1.5% bovine serum albumin (CD68 1:100, β_3 _integrin 1:200 and cathepsin K 1:8000) at 4°C overnight or for 2 hours at room temperature. After thorough rinsing, the sections were incubated with an affinity-purified, biotinylated secondary antibody (1:200 in PBS), followed by incubation with avidin-biotin-peroxidase complex for 30 minutes each, at room temperature. After rinsing, the sections were developed with diaminobenzidine tetrahydrochloride substrate (Vector Laboratoriess, Burlingame, CA USA) and counterstained with hematoxylin, and then sealed with Permount (Fisher Scientific Company, Fair Lawn, NJ, USA). Sections were observed and photographed using a Nikon transmitted light microscope. Routine control experiments for checking specificity of the primary and secondary antibodies were performed by replacing the specific antibody with normal IgG or PBS.

### *In situ *hybridisation

For *in situ *hybridization, RNA sense and antisense probes were transcribed and labeled with ^35^S dATP (New Life Science, Boston, MA, USA) using an *in vitro *transcription kit, as previously described [11,12]. The hybridization solution contained the following: 50% (vol/vol) de-ionized formamide; 10% (weight/vol) dextran sulphate; 1 × Denhardt's solution; 0.02% (weight/vol) of each of bovine serum albumin, Ficoll and polyvinylpyrrolidone, 4 × SSC (sodium chloride and sodium citrate), denatured salmon sperm DNA (0.5 μg/μl) and yeast tRNA (0.25 μg/μl); 1% (weight/vol) sodium *N*-lauroylsarcosinate; and 20,000 counts per minute (cpm) ^35^S-labeled oligonucleotide probe per microliter. Dithiothreitol was directly added at 0.1 mol/l to the hybridization solution before use.

The hybridization procedures used were similar to those used previously [11,12]. Briefly, sections were dewaxed and post-fixed in 4% (weight/vol) freshly prepared paraformadehyde in PBS, acetylated with 0.25% (vol/vol) acetic anhydride in 0.1 mol/l triethanolamine buffer, and then dehydrated in increasing concentrations of ethanol. Each section was hybridized with 10^5 ^cpm labeled sense or antisense RNA probes in a humid chamber overnight at 52°C. After hybridization, the sections were washed in 2 × SSC at 50°C and then dehydrated in an ascending series of ethanol solutions containing 0.3 mol/l ammonium acetate. After dipping in Kodak photographic emulsion, the sections were stored with desiccant at 4°C for 12–20 weeks. The photoemulsion was developed and fixed, and sections were counterstained with hematoxylin and mounted in Kaiser's medium (glycerol/gelatin; Merck, Darmstadt, Germany). The slides were examined and photographed with both bright-field and dark-field illumination.

## Results

**Figure 1:**
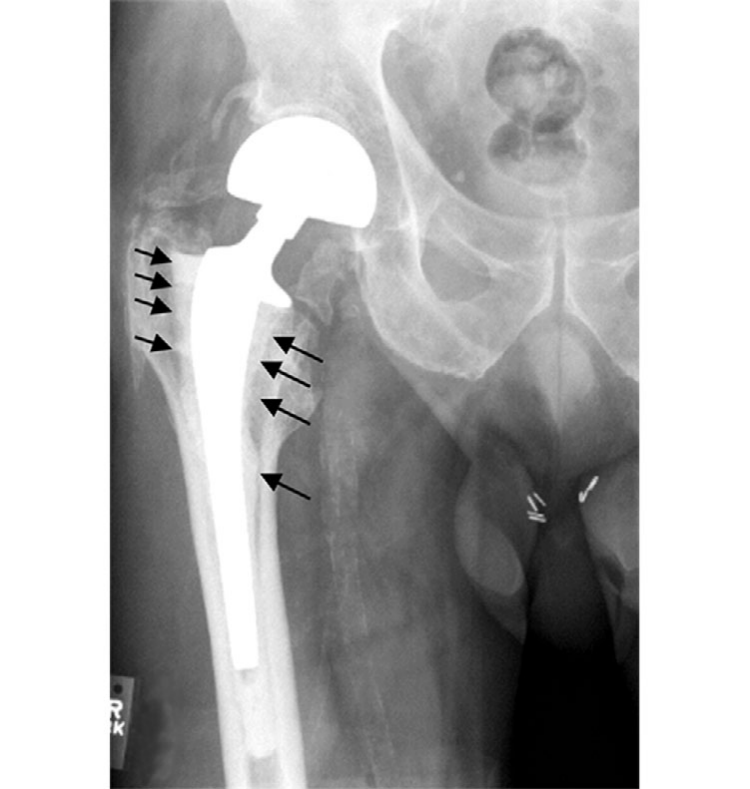
Preoperative radiograph from a study patient before revision hip arthroplasty for aseptic loosening. Arrows denote the area of extensive peri-implant osteolysis along the femoral shaft.

Figure 1 is a representative radiograph from one of the study patients taken before revision hip arthroplasty. The radiograph demonstrates extensive peri-implant osteolysis along the femoral shaft. Tissues from this patient and the other individuals involved in the study were retrieved from the regions of peri-implant osteolysis and assessed for expression of macrophage and osteoclast cell markers.

**Figure 2:**
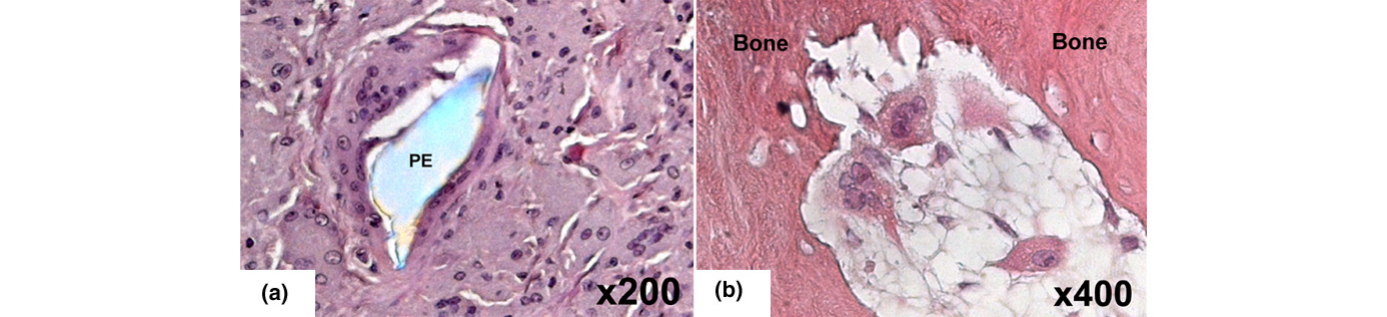
Sections of human peri-implant tissues stained by hematoxylin and eosin. **(a) **Multinucleated cell associated with a polyethylene (PE) wear particle. **(b) **Multinucleated cells line the bone surface at site of bone resorption.

As expected, a chronic granulomatous inflammatory reaction consisting of histiocytes, fibroblasts, and multinucleated foreign body giant cells was present in all specimens. Large numbers of polyethylene particles of varying size (identified by strong birefringence under polarized light microscopy) were distributed throughout the tissues. Many of the larger polyethylene particles were associated with multinucleated foreign body giant cells (Figure 2a). Examination of the interface between the bone and adjacent peri-implant membrane revealed focal regions exhibiting resorption lacunae containing mononucleated and multinucleated osteoclast-like cells (Figure 2b).

**Figure 3:**
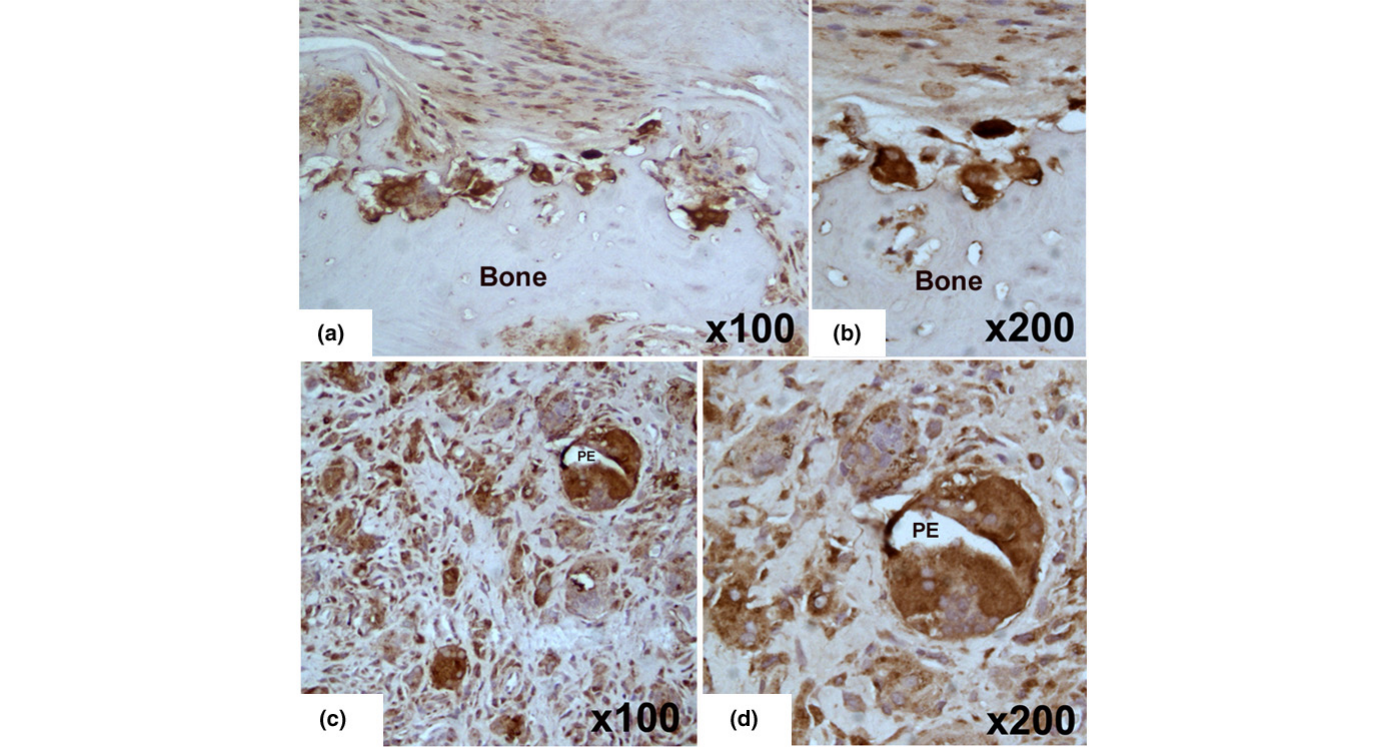
CD68 detection in sections of human peri-implant tissues using immunohistochemistry with rabbit polyclonal antibody. **(a, b) **CD68 is detected in the multinuclear and mononuclear cells located in the soft tissues and multinuclear cells on the bone surface. **(c, d) **CD68 is also detected in multinuclear and mononuclear cells associated with polyethylene (PE) particles.

Previous studies have shown that CD68 is expressed by multiple cell types derived from the CFU-M lineage, including tissue macrophages and osteoclasts [14]. Positive CD68 staining was detected in large numbers of mononucleated and multinucleated cells throughout the membranes. Figure 3a, b shows representative images of the immunohistochemical staining pattern of CD68 seen in the peri-implant tissues. Mononuclear and multinuclear cells present on bone surfaces were strongly positive for CD68. Cells exhibiting a more fibroblastic morphology were CD68 negative. Similar positive staining was detected in mononuclear and multinuclear cells associated with polyethylene particles (Figure 3c, d).

**Figure 4:**
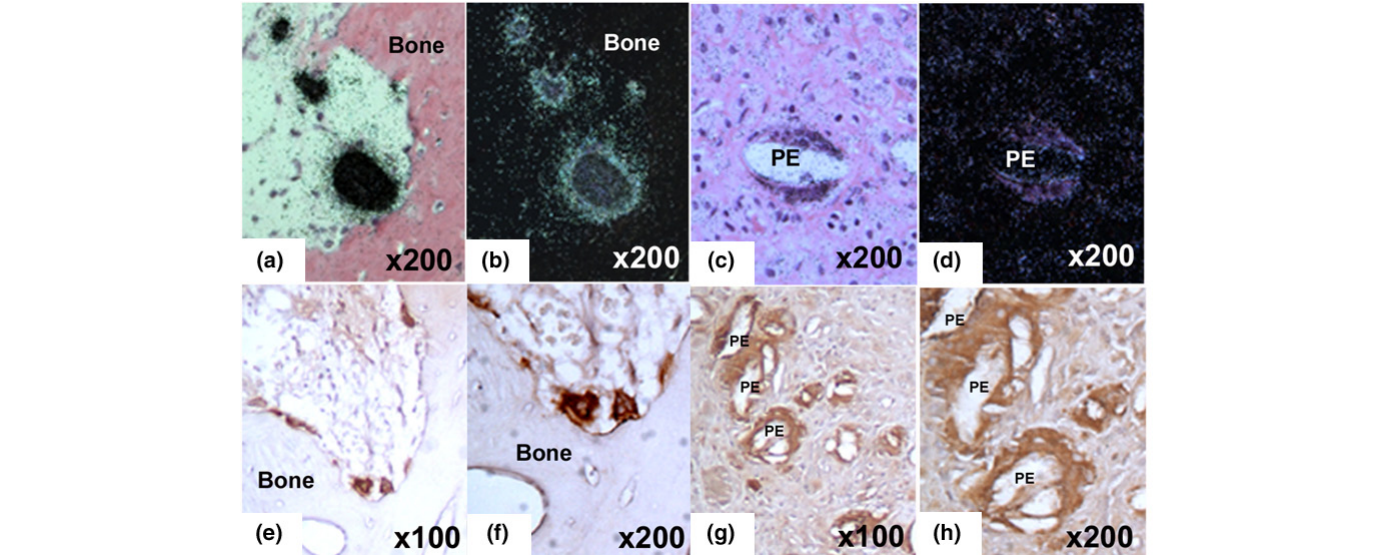
Detection of cathepsin K mRNA and protein in sections of human peri-implantation tissues. The techniques used were *in situ *hybridization with a ^35^S-labeled anti-sense RNA probe (**(a, c) **bright field and **(b, d) **dark field) and **(e-h) **immunohistochemistry with a rabbit polyclonal antibody to human cathepsin K. Multinuclear cells on the bone surface and some mononuclear cells in the peri-implant tissues adjacent to the bone express high levels of cathepsin K mRNA (panels a [bright field] and b [dark field]). Low levels of cathepsin K mRNA were detected in multinuclear cells associated with polyethylene (PE) particles (panels c [bright field] and d [dark field]). Multinuclear cells on the bone surface (panels e and f) and mononuclear and multinuclear cells associated with PE particles (panels g and h) stain positively for cathepsin K protein. PE particles are easily identified by their strong bi-refringence with polarizing light microscopy.

**Figure 5:**
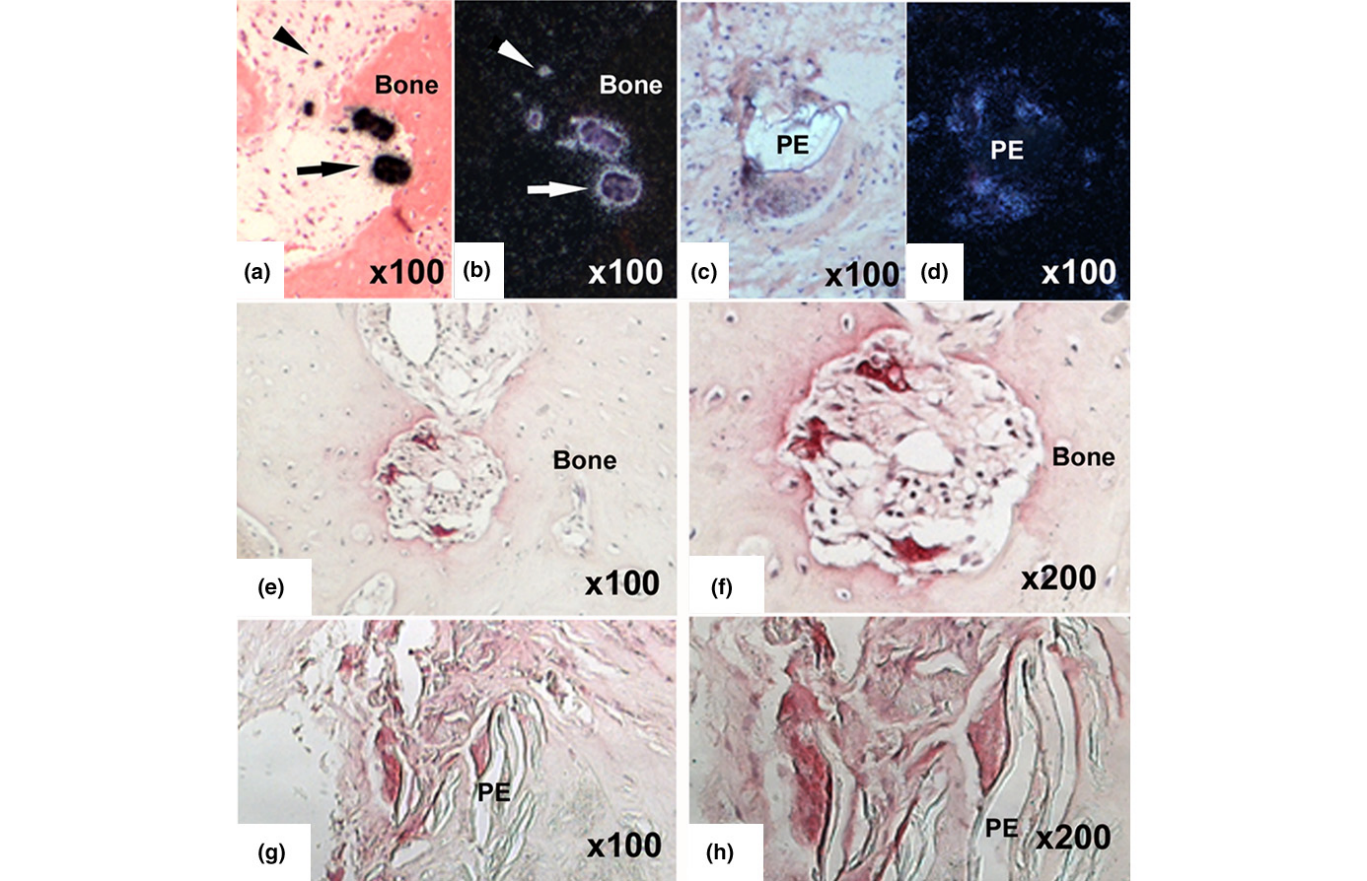
Detection of TRAP mRNA and TRAP enzymatic activity in sections of human peri-implant tissues. The techniques used were *in situ *hybridization with a ^35^S-labeled antisense RNA probe (**(a, c) **bright field and **(b, d) **and dark field) and **(e-h) **histochemistry. TRAP mRNA expression is detected in mononuclear (arrows) and multinuclear cells (arrowheads) on the bone surface adjacent to the peri-implant granuloma (panels a and b). Cells associated with polyethylene (PE) particles also express TRAP mRNA (panels c and d). The enzymatic activity is evident as purple staining seen in similar cells (panels e-h). TRAP, tartrate-resistant acid phosphatase.

*In situ *hybridization and immunohistochemical techniques were used to examine cells for the expression of cathepsin K or TRAP mRNA and protein. These gene products have been used to distinguish osteoclasts from macrophages and other CFU-M lineage cells. As shown in Figure 4a, b, multinucleated cells associated with the bone surface exhibit high levels of expression of cathepsin K mRNA. Surprisingly, mononucleated and multinucleated cells associated with polyethylene particles expressing cathepsin K mRNA were detected in all of the tissues examined, although the mRNA levels were much lower in cells associated with polyethylene than in cells on the bone surfaces (Figure 4c, d). Immunohistochemical staining with an antibody to cathepsin K revealed a similar pattern of cathepsin K protein expression, with differential positive staining between cells associated with polyethylene (Figure 4g, h) and those on the bone (Figure 4e, f). Examination of peri-implant tissues for TRAP mRNA expression revealed a pattern similar to that of cathepsin K. TRAP mRNA was detected in mononuclear and multinuclear cells associated with both the bone surface (Figure 5a, b) and the polyethylene particles (Figures 5c, d), although the message levels were notably higher in the cells located in resorption lacunae at the bone surface. Examination of tissues for the expression of TRAP enzymatic activity revealed similar patterns of differential TRAP activity in cells associated with the polyethylene particles and in those associated with bone surfaces (Figure 5e-h).

**Figure 6:**
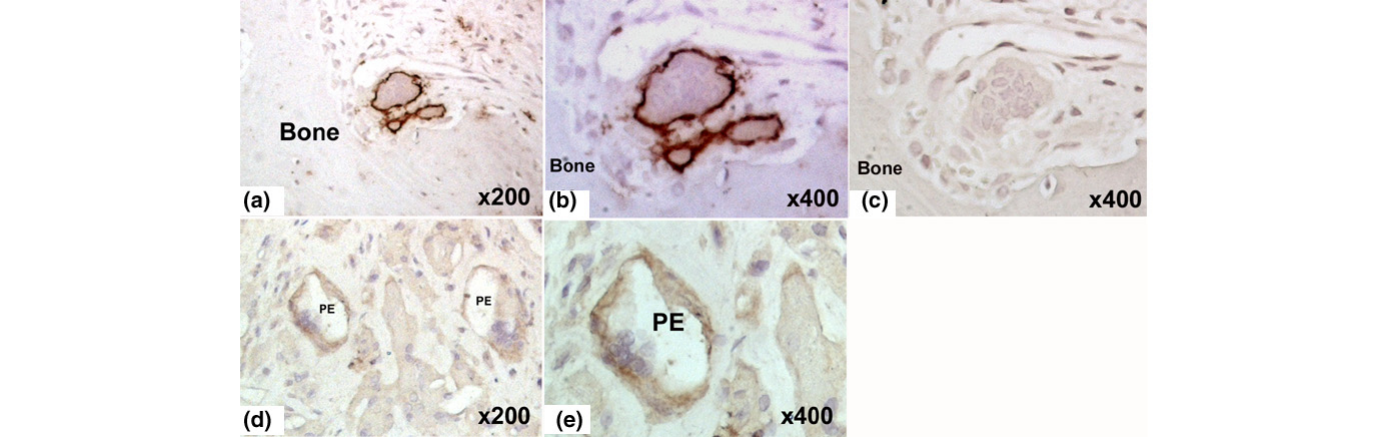
Detection of β_3 _integrin in sections of human peri-implant by immunohistochemistry using a rabbit polyclonal antibody. **(a, b) **β_3 _Integrin is evident in the membrane of mononuclear and multinuclear cells adjacent to bone. **(d, e) **Weak staining is evident in cells associated with polyethylene (PE) particles. Normal IgG was used as a negative control (panel c).

**Figure 7:**
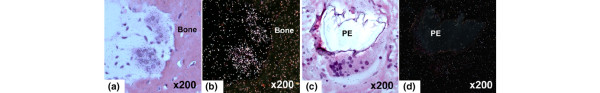
Detection of CTR mRNA in sections of human peri-implant tissues. The techniques used were *in situ *hybridization with a ^35^S-labeled antisense RNA probe (**(a, c) **bright field and **(b, d) **dark field). Expression of CTR mRNA is evident only in multinuclear cells in contact with the bone surface (panels a and b). Cells associated with polyethylene (PE) particles do not express detectable CTR mRNA (panels c and d).

To further characterize cells associated with bone substrates and polyethylene, tissues were examined for β_3 _integrin protein and CTR mRNA expression. As shown in Figure 6a, b, β_3 _integrin immunohistochemical staining was detected in both the mononuclear and multinuclear cells in resorption lacunae on the bone surface. Figure 6c shows negative staining with the secondary antibody alone. Very weak staining was sometimes evident in cells associated with polyethylene particles (Figure 6d, e). In contrast, CTR expression was restricted to multinucleated cells within resorption lacunae (Figure 7a, b). In no instance did we identify cells expressing CTR mRNA associated with the polyethylene particles (Figure 7c, d). These findings suggest that expression of the CTR distinguished osteoclast cells from tissue macrophages and foreign body giant cells, separating it from the other osteoclast cell markers used in this study.

## Discussion

Aseptic loosening of prosthetic joint implants has emerged as the major long-term complication after TJR. The radiographic hallmark of prosthetic loosening is the presence of radiolucent zones at the interface between the bone and adjacent implant materials [15-18]. These zones of osteolysis develop as a consequence of an active biologic process involving the resorption of bone at the peri-implant sites. Insights into the mechanisms involved in this focal disorder of bone remodeling have been provided by histopathologic examination and biochemical analysis of the tissues obtained at revision surgery from patients who have developed aseptic loosening after TJR [17,19-23]. Charnley [24], in his studies of the natural history of patients after total hip replacement, was the first to describe the presence of a 'macrophage foreign body reaction' associated with fragmented methylmethacrylate cement in peri-implant tissues from loosened prostheses. Subsequently, studies have shown that wear particles from orthopedic prosthetic devices of different composition, including polyethylene and metal, have the capacity to induce a foreign body granulomatous reaction [19,20,25-28].

Cells exhibiting phenotypic features of macrophages and macrophage polykaryons (so-called foreign body giant cells) are the principal cell types within the peri-implant granuloma [17,20,21,23,27,28]. In the regions immediately adjacent to the implant surface the cells are frequently organized into a lining layer with histologic features of synovium [20]. Analysis of the granuloma-bone interface has revealed highly variable cellular composition. In many regions the bone is lined by cells that exhibit a fibroblastic morphology. These cells fail to express the CD68 antigen and are not believed to be of hematopoietic origin. The association of the fibroblastic cells with the bone surface and the *ex vivo *demonstration that they can resorb a bone matrix have led some investigators to speculate that these cells represent a connective tissue subtype possessing a unique capacity to resorb bone [29]. In addition to the bone-tissue interface populated by fibroblasts, Willert and coworkers [28] described regions of the bone surface that were lined by multinucleated cells with morphologic features of osteoclasts residing in resorption lacunae. Based on these observations, the conclusion was drawn that the osteolytic process was mediated principally via osteoclastic mechanisms.

Greenfield and coworkers [25] and other investigators [30-32] have suggested that increased recruitment of osteoclast precursors and their differentiation play a major role in wear particle induced osteolysis. To identify the source of the osteoclasts associated with the peri-implant osteolysis, Athanathou's group [14,33,34] and other investigators [35] have isolated cells from peri-implant granulomatous tissue from patients with aseptic loosening and showed that a subset of the mononuclear cells isolated from the tissue could be induced to form bone resorbing osteoclasts when cultured under appropriate conditions. These findings firmly establish the existence of osteoclast precursors within the granuloma of peri-implant tissue. It is presumed that this pool of cells gives rise to the osteoclasts that are associated with the bone surface and mediate the osteolytic process *in vivo*.

Cathepsin K and TRAP are among the enzymes that are expressed by osteoclasts. The importance of these two gene products in osteoclast functional activity is indicated by the resorptive defect in osteoclast-mediated bone resorption in mice in which either of these genes has been deleted [36,37]. In humans, inactivating mutations in the cathepsin K gene are associated with an osteoclast resorbing defect manifest by pycnodysostosis [38]. In the present study we detected cathepsin K and TRAP expression in mononuclear and multinuclear cells associated with polyethylene particles as well as the bone surface. These findings are consistent with the observations reported by Ren and coworkers [39], who found that polyethylene particles also induced cathepsin K and TRAP expression in an air pouch implantation model, and by Konttinen and colleagues [7], who found cathepsin K to be highly expressed in bone resorption around total hip replacement prosthesis. Of importance, in our studies we observed that the levels of expression of both cathepsin K and TRAP were much higher in the cells associated with the bone surface compared with the polyethylene particles. This was evident at the mRNA as well as the protein or functional level. We speculate that interaction with the bone substrate may play a critical role inducing the higher levels of expression of these two enzymes in cells associated with the bone surface, as discussed below.

Although expressions of the cathepsin K and TRAP genes have served as markers of the osteoclast phenotype, under certain conditions cathepsin K and TRAP activity have been detected in cells that are not involved directly in bone resorption [8-10,40], indicating that their expression is not restricted to osteoclasts. For example, macrophages have been shown to express both of these enzymes [41]. In the present study we observed that β_3 _integrin and CTR were preferentially expressed in cells associated with the bone surface. A number of studies have shown that neither the β_3 _integrin nor the CTR are expressed by osteoclast precursors. The expression of these genes increases during the late stages of osteoclast differentiation [42], after induction of the cathepsin K and TRAP genes. Importantly, the transcriptional activation of the β_3 _integrin and CTR genes coincides with the transition of the osteoclast to an actively resorbing cell [10]. Thus, based on these *in vitro *studies, the levels of expression of these genes can be used to discriminate osteoclasts from macrophages or macrophage polykaryons, and to identify osteoclasts that are actively involved in bone resorption. Our results suggest that expression of the CTR may be a more definitive marker of terminal osteoclast differentiation than the β_3 _integrin because it was solely confined to the bone matrix whereas β_3 _was very weakly detected on some multinucleated cells associated with wear particles. Because osteoclast cells express relatively low levels of CTR mRNA, it is also possible, as with the β_3 _integrin, that CTR could be expressed in the cells associated with the polyethylene particles but the levels were below the limits of detection.

Relatively few studies have rigorously compared the functional and phenotypic features of the multinucleated foreign body giant cells associated with wear particles and the multinucleated osteoclast-like cells detected in resorption lacunae at sites of peri-implant osteolysis. Willert and coworkers [28] commented on the absence of wear particles in osteoclast-like cells in resorption lacunae and speculated that they were phenotypically distinct from the polykaryons associated with the implant wear particles. More recently, Athanasou and coworkers [14,43] cultured mononuclear cells isolated from the peri-implant tissues retrieved from patients with granulomatous reactions to implant materials. They showed that both monocyte/macrophages and cells expressing phenotypic features of foreign body giant cells could produce 'resorption pits' on bone slices. The level of resorption produced by these cells, however, was considerably less than that observed with authentic osteoclasts. This suggests that the cells associated with wear particles, despite being multinuclear, are functionally distinct from osteoclasts. These observations support the work of others who have found that macrophages or macrophage polykaryons have a limited capacity to resorb a mineralized bone matrix [26,27,44].

Further evidence indicating the differential phenotype of foreign body giant cells and osteoclast cells is provided by our previous studies [45,46] in which we implanted particles of polyethylene or polymethylmethacrylate into soft tissues of rats and analyzed the phenotypic features of the elicited cells. We observed that the particulate polymeric materials failed to induce cells with the full phenotypic and functional properties of osteoclasts. In these studies we also implanted mineralized bone particles of size comparable to that of the polyethylene and polymethylmethacrylate particles. The bone, similar to the polymeric materials, induced a granulomatous inflammatory reaction. However, in contrast to the polymeric material, the bone particles induced multiunucleated cells expressing TRAP activity as well as CTR. Most importantly, these cells were able to resorb the bone matrix, thus establishing their authenticity as osteoclast cells. These observations, and the findings of the present study using human peri-implant tissues, indicate that binding of cells to polyethylene wear particles or bone results in differential phenotypic expression. Based on these findings we speculate that binding and interaction of cells with the different substrates play important roles in regulating their differential phenotypic and functional activity.

In the present study we noted the presence of abundant CD68 positive mononucleated and multinucleated cells associated with the polyethylene particles. Cells within resorption lacunae at the bone surface also expressed this antigen. Other studies have shown that osteoclasts, as well as CFU-M lineage macrophages, express CD68, and we speculate that the CD68-positive mononuclear cells within the granuloma represent the precursors for the osteoclast-like cells involved in the resorptive process at the bone surface. This conclusion is supported by recent studies conducted by Sabokbar and coworkers [14], who demonstrated that macrophages isolated directly from peri-implant tissues surrounding loosened implants exhibited limited capacity to resorb bone. However, under appropriate culture conditions they could be induced *ex vivo *to differentiate into multinucleated cells with the full functional and cytochemical characteristics of osteoclasts. These findings received support from a study conducted by Haynes and coworkers [35].

## Conclusion

Our results provide further evidence implicating osteoclasts in the pathogenesis of the peri-implant osteolysis associated with prosthetic implant wear debris. The findings of TRAP and cathepsin K expression in macrophages and foreign body giant cells associated with wear particles expression confirms our previous observations, as well as the findings of others, that these two enzymes are not definitive markers of the osteoclast phenotype [8-10,41]. β_3 _Integrin and CTR are associated with later stage cells of osteoclast differentiation, and their levels of expression were much high in cells in contact with bone as opposed to wear particles. The expression of the CTR appears to discriminate osteoclasts from foreign body giant cells and other CFU-M lineage cells because it is expressed solely by cells on the bone. The findings supporting a role for osteoclasts in the pathogenesis of peri-implant osteolysis have important clinical implications and suggest that targeting osteoclasts, as well as the pathways that regulate osteoclast differentiation and activation, represents a rational therapeutic approach to preventing this major clinical complication after TJR.

## Abbreviations

CFU-M = colony forming units-macrophage; CTR = calcitonin receptor; counts per minute (cpm); PBS = phosphate-buffered saline; TJR = total joint replacement; TRAP = tartrate resistant acid phosphatase.

## Competing interests

The authors declare that they have no competing interests.

## Authors' contributions

ZS and SRG participated in the design of the study. BEB provided surgical samples. ZS performed histochemisty, immunohischemistry, and *in situ *hybridization assisted by EMG and KM. ZS and TNC prepared the figures and drafted the manuscript. SRG, KPM and EMG reviewed the manuscript. All authors read and approved the final manuscript.

## References

[B1] Takagi M, Santavirta S, Ida H, Ishii M, Takei I, Niissalo S, Ogino T, Konttinen YT (2001). High-turnover periprosthetic bone remodeling and immature bone formation around loose cemented total hip joints. J Bone Miner Res.

[B2] Shinar DM, Schmidt A, Halperin D, Rodan GA, Weinreb M (1993). Expression of alpha v and beta 3 integrin subunits in rat osteoclasts *in situ*. J Bone Miner Res.

[B3] Davies J, Warwick J, Totty N, Philp R, Helfrich M, Horton M (1989). The osteoclast functional antigen, implicated in the regulation of bone resorption, is biochemically related to the vitronectin receptor. J Cell Biol.

[B4] Nesbitt S, Nesbit A, Helfrich M, Horton M (1993). Biochemical characterization of human osteoclast integrins. Osteoclasts express alpha v beta 3, alpha 2 beta 1, and alpha v beta 1 integrins. J Biol Chem.

[B5] Teitelbaum S (2000). Bone resorption by osteoclasts. Science.

[B6] Troen BR (2004). The role of cathepsin K in normal bone resorption. Drug News Perspect.

[B7] Konttinen YT, Takagi M, Mandelin J, Lassus J, Salo J, Ainola M, Li TF, Virtanen I, Liljestrom M, Sakai H (2001). Acid attack and cathepsin K in bone resorption around total hip replacement prosthesis. J Bone Miner Res.

[B8] Athanasou NA (1996). Cellular biology of bone-resorbing cells. J Bone J Surg Am.

[B9] Hattersley G, Chambers TJ (1989). Generation of osteoclastic function in mouse bone marrow cultures: multinuclearity and tartrate-resistant acid phosphatase are unreliable markers for osteoclastic differentiation. Endocrinology.

[B10] Chambers TJ (2000). Regulation of the differentiation and function of osteoclasts. J Pathol.

[B11] Gravallese EM, Harada Y, Wang JT, Gorn AH, Thornhill TS, Goldring SR (1998). Identification of cell types responsible for bone resorption in rheumatoid arthritis and juvenile rheumatoid arthritis. Am J Pathol.

[B12] Gravallese EM, Manning C, Tsay A, Naito A, Pan C, Amento E, Goldring SR (2000). Synovial tissue in rheumatoid arthritis is a source of osteoclast differentiation factor. Arthritis Rheum.

[B13] Shen Z, Heinegard D, Sommarin Y (1995). Distribution and expression of cartilage oligomeric matrix protein and bone sialoprotein show marked changes during rat femoral head development. Matrix Biol.

[B14] Sabokbar A, Fujikawa Y, Neale S, Murray DW, Athanasou NA (1997). Human arthroplasty derived macrophages differentiate into osteoclastic bone resorbing cells. Ann Rheum Dis.

[B15] Gruen TA, McNeice GM, Amstutz HC (1979). 'Modes of failure' of cemented stem-type femoral components: a radiographic analysis of loosening. Clin Orthop Relat Res.

[B16] Zicat B, Engh CA, Gokcen E (1995). Patterns of osteolysis around total hip components inserted with and without cement. J Bone Joint Surg Am.

[B17] Santavirta S, Hoikka V, Eskola A, Konttinen YT, Paavilainen T, Tallroth K (1990). Aggressive granulomatous lesions in cementless total hip arthroplasty. J Bone Joint Surg Br.

[B18] Clohisy JC, Harris WH (1999). Primary hybrid total hip replacement, performed with insertion of the acetabular component without cement and a precoat femoral component with cement. An average ten-year follow-up study. J Bone Joint Surg Am.

[B19] Goldring SR, Jasty M, Roelke MS, Rourke CM, Bringhurst FR, Harris WH (1986). Formation of a synovial-like membrane at the bone-cement interface. Its role in bone resorption and implant loosening after total hip replacement. Arthritis Rheum.

[B20] Goldring SR, Schiller AL, Roelke M, Rourke CM, O'Neil DA, Harris WH (1983). The synovial-like membrane at the bone-cement interface in loose total hip replacements and its proposed role in bone lysis. J Bone Joint Surg [Am].

[B21] Cook SD, McCluskey LC, Martin PC, Haddad RJ (1991). Inflammatory response in retrieved noncemented porous-coated implants. Clin Orthop Relat Res.

[B22] Schmalzried TP, Kwong LM, Jasty M, Sedlacek RC, Haire TC, O'Connor DO, Bragdon CR, Kabo JM, Malcolm AJ, Harris WH (1992). The mechanism of loosening of cemented acetabular components in total hip arthroplasty. Analysis of specimens retrieved at autopsy. Clin Orthop Relat Res.

[B23] Jiranek WA, Machado M, Jasty M, Jevsevar D, Wolfe HJ, Goldring SR, Goldberg MJ, Harris WH (1993). Production of cytokines around loosened cemented acetabular components. Analysis with immunohistochemical techniques and *in situ* hybridization. J Bone Joint Surg Am.

[B24] Charnley J (1961). Arthroplasty of the hip. A new operation. Lancet.

[B25] Greenfield EM, Bi Y, Ragab AA, Goldberg VM, Van De Motter RR (2002). The role of osteoclast differentiation in aseptic loosening. J Orthop Res.

[B26] Haynes DR, Crotti TN, Zreiqat H (2004). Regulation of osteoclast activity in peri-implant tissues. Biomaterials.

[B27] Ingham E, Fisher J (2005). The role of macrophages in osteolysis of total joint replacement. Biomaterials.

[B28] Willert HG, Buchhorn GH, Hess T (1989). The significance of wear and material fatigue in loosening of hip prostheses [in German]. Orthopade.

[B29] Pap T, Claus A, Ohtsu S, Hummel KM, Schwartz P, Drynda S, Pap G, Machner A, Stein B, George M (2003). Osteoclast-independent bone resorption by fibroblast-like cells. Arthritis Res Ther.

[B30] Eftekhar NS, Doty SB, Johnston AD, Parisien MV (1985). Prosthetic synovitis. Hip.

[B31] Friedman RJ, Black J, Galante JO, Jacobs JJ, Skinner HB (1994). Current concepts in orthopaedic biomaterials and implant fixation. Instr Course Lect.

[B32] Kadoya Y, Revell PA, al-Saffar N, Kobayashi A, Scott G, Freeman MA (1996). Bone formation and bone resorption in failed total joint arthroplasties: histomorphometric analysis with histochemical and immunohistochemical technique. J Orthop Res.

[B33] Neale S, Sabokbar A, Howie DW, Murray DW, Athanasou NA (1999). Macrophage colony-stimulating factor and interleukin-6 release by periprosthetic cells stimulates osteoclast formation and bone resorption. J Orthop Res.

[B34] Neale SD, Athanasou NA (1999). Cytokine receptor profile of arthroplasty macrophages, foreign body giant cells and mature osteoclasts. Acta Orthop Scand.

[B35] Haynes DR, Crotti TN, Potter AE, Loric M, Atkins GJ, Howie DW, Findlay DM (2001). The osteoclastogenic molecules RANKL and RANK are associated with periprosthetic osteolysis. J Bone Joint Surg Br.

[B36] Gowen M, Lazner F, Dodds RA, Kapadia R, Feild J, Tavaria M, Bertoncello I, Drake FH, Zavarselk S, Tellis I (1999). Cathepsin K knock-out mice develop osteopetrosis due to a deficit in matrix degradation but not mineralization. J Bone Min Res.

[B37] Hayman AR, Jones SJ, Boyde A, Foster D, Colledge WH, Carlton MB, Evans MJ, Cox TM (1996). Mice lacking tartrate-resistant acid phosphatase (Acp 5) have disrupted endochondral ossification and mild osteopetrosis. Development.

[B38] Gelb BD, Shi GP, Chapman HA, Desnick RJ (1996). Pycnodysostosis, a lysosomal disease caused by cathepsin K deficiency. Science.

[B39] Ren W, Yang SY, Fang HW, Hsu S, Wooley PH (2003). Distinct gene expression of receptor activator of nuclear factor-kappaB and rank ligand in the inflammatory response to variant morphologies of UHMWPE particles. Biomaterials.

[B40] Mandelin J, Li TF, Hukkanen M, Liljestrom M, Salo J, Santavirta S, Konttinen YT (2005). Interface tissue fibroblasts from loose total hip replacement prosthesis produce receptor activator of nuclear factor-kappaB ligand, osteoprotegerin, and cathepsin K. J Rheumatol.

[B41] Mandelin J, Liljestrom M, Li TF, Ainola M, Hukkanen M, Salo J, Santavirta S, Konttinen YT (2005). Pseudosynovial fluid from loosened total hip prosthesis induces osteoclast formation. J Biomed Mater Res B Appl Biomater.

[B42] Inoue M, Namba N, Chappel J, Teitelbaum SL, Ross FP (1998). Granulocyte macrophage-colony stimulating factor reciprocally regulates alphav-associated integrins on murine osteoclast precursors. Mol Endocrinol.

[B43] Neale SD, Sabokbar A, Fujikawa Y, Howie DW, Graves SE, Murray DW, Athanasou NA (1999). Human bone stromal cells support osteoclast formation from arthroplasty-derived cells in vitro. Evidence for prostaglandin stimulation of bone resorption. SIROT.

[B44] Murray DW, Rushton N (1990). Macrophages stimulate bone resorption when they phagocytose particles. J Bone Joint Surg Br.

[B45] Glowacki J, Jasty M, Goldring S (1986). Comparison of multinucleated cells elicited in rats by particulate bone, polyethylene, or polymethylmethacrylate. J Bone Miner Res.

[B46] Goldring SR, Roelke M, Glowacki J (1988). Multinucleated cells elicited in response to implants of devitalized bone particles possess receptors for calcitonin. J Bone Miner Res.

